# National Trends in Utilization and In-Hospital Outcomes of Surgical Aortic Valve Replacements in Spain, 2001-2015

**DOI:** 10.21470/1678-9741-2019-0181

**Published:** 2020

**Authors:** Rodrigo Jiménez-García, Napoleón Perez-Farinos, Javier de Miguel-Díez, Valentín Hernández-Barrera, Manuel Méndez-Bailón, Isabel Jimenez-Trujillo, José M. de Miguel-Yanes, Ana López-de-Andrés

**Affiliations:** 1 Preventive Medicine and Public Health Teaching and Research Unit, Health Sciences Faculty, Rey Juan Carlos University, Madrid, Spain.; 2 Department of Public Health and Psychiatry, Faculty of Medicine, Universidad de Málaga, Andalucía, Spain.; 3 Respiratory Department, Hospital General Universitario Gregorio Marañón, Universidad Complutense de Madrid, Instituto de Investigación Sanitaria Gregorio Marañón, Madrid, Spain.; 4 Internal Medicine Department, Hospital Universitario Clínico San Carlos, Universidad Complutense de Madrid, Madrid, Spain.; 5 Internal Medicine Department, Hospital General Gregorio Marañon, Madrid, Spain.

**Keywords:** Aortic Valve, Hospital Mortality, Incidence, Spain, Heart Valve Prosthesis, Coronary Artery Bypass, Pacemarker Artificial

## Abstract

**Objective:**

The aims of this study were to examine the incidence and in-hospital outcomes of surgical aortic valve replacement (SAVR) and to identify factors associated with in-hospital mortality (IHM) among patients according to the type of implanted valve used in SAVR.

**Methods:**

We performed a retrospective study using the Spanish National Hospital Discharge Database, 2001-2015. We included patients who had SAVR listed as a procedure in their discharge report.

**Results:**

We identified 86,578 patients who underwent SAVR (52.78% mechanical and 47.22% bioprosthetic). Incidence of SAVR coding increased significantly from 11.95 cases per 100,000 inhabitants in 2001 to 17.92 in 2015 (*P*<0.001). Age and comorbidities increased over time (*P*<0.001). There was a significant increase in the frequency of concomitant coronary artery bypass grafting (CABG) and in the use of pacemaker implantation. The use of mechanical SAVR decreased and the use of bioprosthetic valves increased over time. IHM decreased over time (from 8.13% in 2001-05 to 5.39% in 2011-15). Patients who underwent mechanical SAVR had higher IHM than those who underwent bioprosthetic SAVR (7.44% *vs*. 6%; *P*<0.05). Higher IHM rates were associated with advanced age, female sex, comorbidities, concomitant CABG, and the use of mechanical SAVR (OR 1.67; 95% CI 1.57-1.77).

**Conclusion:**

The number of SAVRs performed in Spain has increased since 2001. The use of mechanical SAVR has decreased and the use of bioprosthetic valves has increased over time. IHM has decreased over time for both types of valves and despite a concomitant increase in age and comorbidities of patients during the same period.

**Table:** 

Abbreviations, acronyms & symbols				
ANOVA	= Analysis of variance		NIS	= National Inpatient Sample
CABG	= Coronary artery bypass grafting		OR	= Odds ratio
CCI	= Charlson comorbidity index		PCS	= Previous cardiac surgery
COPD	= Chronic obstructive pulmonary disease		SAVR	= Surgical aortic valve replacement
ER	= Emergency room		SNHDD	= Spanish National Hospital Discharge Database
IHM	= In-hospital mortality		TAVR	= Transcatheter aortic valve replacement
LOHS	= Length of hospital stay		T2DM	= Type 2 diabetes mellitus

## INTRODUCTION

Aortic valve replacement is the treatment of choice in severe symptomatic aortic valve disease. Surgical aortic valve replacement (SAVR) reduces mortality, provides symptom relief and increases quality of life at subsequent follow-up^[1]^. Furthermore, continuous improvements in SAVR techniques and new technologies have recently been developed to facilitate the procedure and reduce operative times^[2]^.

The range of available prostheses changed significantly during the last decades in favor of biological valve *versus* mechanical valve replacements^[3]^. A study using the National Inpatient Sample (NIS) found an increase in the use of bioprosthetic valves from 37.7% in 1998-2001 to 63.6% in 2007-2011^[4]^. Biological valves are increasingly implanted in younger patients due to a higher durability enabled by improved anticalcification treatment and the adverse events associated with mechanical prostheses^[4,5]^.

Previous studies have provided insight into changes in patient demographics, risk factors and outcomes of SAVR^[2]^. Therefore, it is necessary to study changes in practice over time and to establish outcomes after SAVR to help inform decision making for high-risk patients^[3]^. However, valve surgery trends and in-hospital outcomes nationwide are less often reported^[6]^.

Using the Spanish National Hospital Discharge Database (SNHDD), this study aimed to: i) examine trends in the incidence, characteristics and in-hospital outcomes of SAVR hospitalizations from 2001 to 2015; ii) compare clinical variables among patients according to the type of implanted valves in the discharge report; and iii) identify factors associated with in-hospital mortality (IHM) among patients according to the type of implanted valves.

## METHODS

This retrospective observational study was performed using the SNHDD. Details of the SNHDD design and description are available online^[7]^.

We selected admitted patients (aged ≥18 years) whose medical procedures included mechanical and bioprosthetic SAVR (ICD-9-CM codes: 35.21,35.22). Patients undergoing one or more additional cardiac procedures (defined as mitral, tricuspid or pulmonic valve replacement, repair or valvulotomy; ascending aorta replacement; closure of ventricular and atrial septal defects; or ablation and other rare procedures) were excluded. We collected data between January 1, 2001 and December 31, 2015.

Clinical characteristics included information on overall comorbidity at the time of discharge, assessed by calculating the Charlson comorbidity index (CCI)^[8]^.

ICD-9-CM codes for conditions included in the CCI, as well as other diseases and procedures performed during the hospital stay and analyzed in this investigation, are shown in Supplementary Table 1.

**Supplementary Table 1:** Diagnosis and procedures with corresponding ICD-9-CM codes

	ICD-9-CM codes
COPD	490, 491, 491.0, 491.1, 491.2x, 491.8, 491.9, 492, 492.0, 492.8, 496
T2DM	250.x0 and 250.x2
Peripheral vascular disease	0.93.0, 473.3, 440.x, 441.x, 443.1-443.9, 447.1, 557.1, 557.9, V43.4
Renal disease	403.01, 403.11, 403.91, 404.02, 404.03, 404.12, 404.13, 404.92, 404.93, 582, 583.0-583.7, 585, 586, 588.0, V42.0, V45.1, V56
Cerebrovascular disease	362.34, 430.x-438.x
Congestive heart failure	398.91, 402.01, 402.11, 402.91, 404.01, 404.03, 404.11, 404.13, 404.91, 404.93, 425.4-425.9, 428.x
Atrial fibrillation	427.31
Pulmonary hypertension	416.0 and 416.8
Coronary artery disease	410.xx, 412.x, 413.x, 414.0, 414, 414.00, 414.01, 414.2-9
CABG	36.10-36.19
Pacemaker implantation	37.70-37.74, 37.80-37.83

CABG=coronary artery bypass grafting; COPD=chronic obstructive pulmonary disease; T2DM=type 2 diabetes mellitus

We evaluated the mean length of hospital stay (LOHS), and estimated the proportion of patients admitted through the emergency room (ER).

The main endpoints in our investigation were trends in the incidence rates of hospitalizations and IHM in patients whose medical procedure was mechanical or bioprosthetic SAVR. IHM was defined by the proportion of patients who died during admission for each year of study.

We considered three time periods that included five consecutive years each (2001-05; 2006-10; 2011-15). We estimate the incidence rates of admission for SAVR calculated per 100,000 inhabitants, according to data from the Spanish National Institute of Statistics^[9]^. Trends in the incidences were assessed using Poisson regression models adjusted by sex and age, when appropriate.

A descriptive statistical analysis was performed for all continuous variables and categories. Variables are expressed as proportions as means with standard deviations. A bivariable analysis according to year was performed using the χ^2^ test for linear trend (proportions) and ANOVA (means), as appropriate.

To identify variables associated with IHM as a binary outcome among patients who underwent SAVR, we performed three logistic regression analyses, one for each type of SAVR. The variables included in the multivariable models were those with significant results in the bivariable analysis and those considered relevant in other investigations. Estimates were the odds ratio (OR) with a 95% confidence interval (95% CI).

All statistical analysis was performed with Stata version 10.1 (Stata, College Station, Texas, USA). Statistical significance was set at *P*<0.05 (two-tailed).

## RESULTS

There were 86,578 hospitalizations of patients who underwent SAVR between 2001 and 2015. We identified 45,697 (52.78%) hospitalized patients who underwent mechanical SAVR and 40,881 (47.22%) who underwent bioprosthetic SAVR.

### Trends in SAVR Hospitalizations

We found that the incidence of SAVR coding increased from 11.95 cases per 100,000 inhabitants in 2001 to 17.92 cases in 2015 (*P*<0.001) (Supplementary Figure 1A). Patient age increased significantly over time, from 67.68±10.94 years in 2001-5 to 70.92±10.85 years in 2011-15. An analysis of sex distribution showed a slight increase in the percentage of women over the study period (39.82% *vs*. 40.15%; *P*=0.041) (Table 1). We detected a significant increase in comorbidity according to the CCI over time. The most common associated comorbidities for hospitalized patients who underwent SAVR were coronary artery disease (32.56%), atrial fibrillation (32.31%) and diabetes (22.9%). The frequency of all conditions analyzed increased over time (*P*<0.001), with the exception of chronic obstructive pulmonary disease (COPD), which showed a slight decrease over the study period (9.1% to 8.88%; *P*=0.011) (Table 1).

**Table 1:** Sociodemographic and clinical characteristics of patients hospitalized who underwent a surgical aortic valve replacement (SAVR) in Spain from 2001 to 2015.

	2001-05	2006-10	2011-15	Total	Trend
Number of SAVRs	22966	28849	34763	86578	
Age, mean (SD)	67.68 (10.94)	69.49 (10.99)	70.92 (10.85)	69.58 (11)	<0.001
18-59 age group, n (%)	4330 (18.85)	4696 (16.28)	4762 (13.7)	13788 (15.93)	<0.001
60-69 age group, n (%)	6429 (27.99)	6759 (23.43)	7731 (22.24)	20919 (24.16)	<0.001
70-79 age group, n (%)	10466 (45.57)	13402 (46.46)	15123 (43.5)	38991 (45.04)	<0.001
≥80 age group, n (%)	1741 (7.58)	3992 (13.84)	7147 (20.56)	12880 (14.88)	<0.001
Female sex, n (%)	9145 (39.82)	11545 (40.02)	13957 (40.15)	34647 (40.02)	0.041
CCI index, mean (SD)	0.82 (0.89)	0.95 (0.94)	1.04 (0.99)	0.95 (0.95)	<0.001
T2DM, n (%)	4060 (17.68)	6619 (22.94)	9148 (26.32)	19827 (22.9)	<0.001
COPD, n (%)	2089 (9.1)	2667 (9.24)	3087 (8.88)	7843 (9.06)	0.011
Peripheral vascular disease, n (%)	2910 (12.67)	4324 (14.99)	5346 (15.38)	12580 (14.53)	<0.001
Chronic kidney disease, n (%)	1168 (5.09)	2090 (7.24)	3431 (9.87)	6689 (7.73)	<0.001
Cerebrovascular disease, n (%)	806 (3.51)	1143 (3.96)	1876 (5.4)	3825 (4.42)	<0.001
Congestive heart failure, n (%)	3309 (14.41)	4363 (15.12)	5739 (16.51)	13411 (15.49)	<0.001
Atrial fibrillation, n (%)	6782 (29.53)	9245 (32.05)	11944 (34.36)	27971 (32.31)	<0.001
Coronary artery disease, n (%)	7100 (30.92)	9611 (33.31)	11482 (33.03)	28193 (32.56)	<0.001
Pulmonary hypertension, n (%)	1232 (5.36)	1779 (6.17)	2109 (6.07)	5120 (5.91)	<0.001
Mechanical valve, n (%)	15582 (67.85)	15001 (52)	15114 (43.48)	45697 (52.78)	<0.001
Bioprosthetic valve, n (%)	7384 (32.15)	13848 (48)	19649 (56.52)	40881 (47.22)	<0.001
CABG, n (%)	4745 (20.66)	6508 (22.56)	7453 (21.44)	18706 (21.61)	<0.001
Pacemaker implantation, n (%)	733 (3.19)	997 (3.46)	1549 (4.46)	3279 (3.79)	<0.001
Emergency room, n (%)	5309 (23.12)	6173 (21.4)	7098 (20.42)	18580 (21.46)	<0.001
LOHS, mean (SD)	20.75 (17.47)	19.73 (17.33)	16.84 (15.83)	18.84 (16.86)	<0.001
IHM, n (%)	1866 (8.13)	2108 (7.31)	1875 (5.39)	5849 (6.76)	<0.001

CABG=coronary artery bypass grafting; CCI=Charlson comorbidity index; COPD=chronic obstructive pulmonary disease; LOHS=length of hospital stay; IHM=in-hospital mortality; T2DM=type 2 diabetes mellitus

**Supplementary Fig. 1A:**
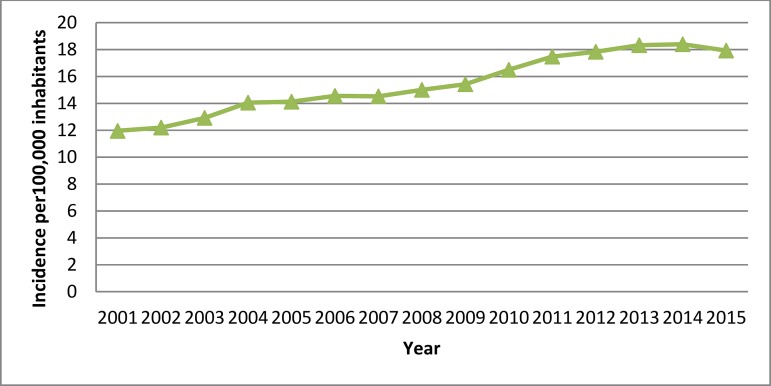
Time trend 2001-2015 in the incidence of hospital admissions for SAVR in Spain.

There was a significant increase in the frequency of concomitant coronary artery bypass grafting (CABG) (from 20.66% in 2001-05 to 21.44% in 2011-15) and in the use of pacemaker implantation (from 3.19% in 2001-05 to 4.46% in 2011-15; *P*<0.001) (Table 1).

The mean LOHS for patients undergoing SAVR was 20.75±17.47 days in 2001-05, decreasing to 16.84±15.83 days in 2011-15 (*P*<0.001). The proportion of patients admitted through the ER also decreased significantly (*P*<0.001) during the study period, from 23.12% in 2001-05 to 20.42% in 2011-15.

For the total period, crude IHM was 6.76%. IHM decreased (*P*<0.001) over time from 8.13% in 2001-05 to 5.39% in 2011-15.

### Trends in Mechanical SAVR Hospitalizations

Incidence rates of hospital admissions with a mechanical SAVR-coded procedure decreased significantly from 8.94 cases per 100,000 inhabitants in 2001 to 7.23 in 2015 (Supplementary Figure 1B). The mean age increased slightly, but significantly, from 65.05 years to 65.5 years, and the percentage of female patients showed a significant decrease over the study period (37.84% in 2001-05 *vs*. 36.09% in 2011-15) (Table 2).

**Supplementary Fig. 1B:**
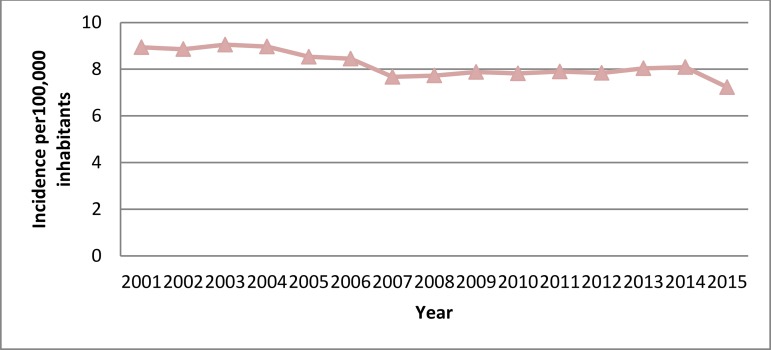
Time trend 2001-2015 in the incidence of hospital admissions for mechanical SAVR in Spain.

**Table 2:** Sociodemographic and clinical characteristics of patients hospitalized who underwent a mechanical surgical aortic valve replacement (SAVR) in Spain from 2001 to 2015.

	2001-05	2006-10	2011-15	Total	Trend
Number of SAVRs	15582	15001	15114	45697	
Age, mean (SD)	65.06 (11.25)	65.25 (11.66)	65.5 (11.99)	65.27 (11.64)	<0.001
18-59 age group, n (%)	4008 (25.72)	4108 (27.38)	4107 (27.17)	12223 (26.75)	0.001
60-69 age group, n (%)	5290 (33.95)	4803 (32.02)	4977 (32.93)	15070 (32.98)	<0.001
70-79 age group, n (%)	5595 (35.91)	4974 (33.16)	4425 (29.28)	14994 (32.81)	<0.001
≥80 age group, n (%)	689 (4.42)	1116 (7.44)	1605 (10.62)	3410 (7.46)	0.001
Female sex, n (%)	5896 (37.84)	5433 (36.22)	5454 (36.09)	16783 (36.73)	0.002
CCI index, mean (SD)	0.81 (0.88)	0.93 (0.93)	1.04 (0.99)	0.92 (0.94)	<0.001
T2DM, n (%)	2617 (16.8)	3201 (21.34)	3711 (24.55)	9529 (20.85)	<0.001
COPD, n (%)	1346 (8.64)	1303 (8.69)	1267 (8.38)	3916 (8.57)	<0.001
Peripheral vascular disease, n (%)	2124 (13.63)	2655 (17.7)	2988 (19.77)	7767 (17)	<0.001
Chronic kidney disease, n (%)	747 (4.79)	958 (6.39)	1197 (7.92)	2902 (6.35)	<0.001
Cerebrovascular disease, n (%)	514 (3.3)	525 (3.5)	725 (4.8)	1764 (3.86)	<0.001
Congestive heart failure, n (%)	2219 (14.24)	2238 (14.92)	2495 (16.51)	6952 (15.21)	<0.001
Atrial fibrillation, n (%)	4398 (28.22)	4367 (29.11)	4617 (30.55)	13382 (29.28)	<0.001
Coronary artery disease, n (%)	4547 (29.18)	4441 (29.6)	4307 (28.5)	13295 (29.09)	0.154
Pulmonary hypertension, n (%)	833 (5.35)	903 (6.02)	944 (6.25)	2680 (5.86)	0.020
CABG, n (%)	2947 (18.91)	2906 (19.37)	2679 (17.73)	8532 (18.67)	<0.001
Pacemaker implantation, n (%)	466 (2.99)	503 (3.35)	634 (4.19)	1603 (3.51)	<0.001
Emergency room, n (%)	3654 (23.45)	3246 (21.64)	3044 (20.14)	9944 (21.76)	<0.001
LOHS, mean (SD)	20.77 (17.54)	19.84 (17.09)	16.88 (16.37)	19.17 (17.09)	<0.001
IHM, n (%)	1316 (8.45)	1191 (7.94)	891 (5.9)	3398 (7.44)	<0.001

CABG=coronary artery bypass grafting; CCI=Charlson comorbidity index; COPD=chronic obstructive pulmonary disease; LOHS=length of hospital stay; IHM=in-hospital mortality; T2DM=type 2 diabetes mellitus

Comorbidity increased significantly over the study period. The prevalence of chronic conditions and use of procedures among patients who underwent mechanical SAVR agreed with the results found in the total sample, except for the prevalence of concomitant CABG, which showed a significant decrease (18.91% to 17.73%; *P*<0.001).

The crude IHM decreased significantly from 8.45% to 5.9% over the study period.

### Trends in Bioprosthetic SAVR Hospitalizations

Supplementary Figure 1C shows a significant and constant increase in the hospitalization rates over time (from 3.01 cases per 100,000 inhabitants in 2001 to 10.7 cases in 2015) for patients who underwent bioprosthetic SAVR.

**Supplementary Fig. 1C:**
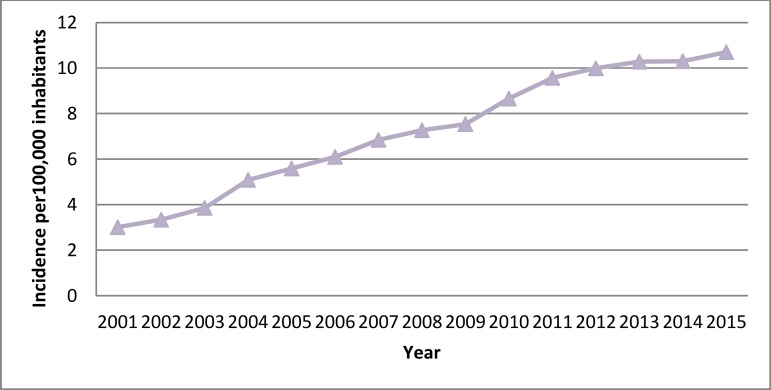
Time trend 2001-2015 in the incidence of hospital admissions for bioprosthetic SAVR in Spain.

The evolution over time of the demographic and clinical variables analyzed was similar to those described for the total sample. The IHM decreased significantly from 7.45% to 5.01% (*P*<0.001) (Table 3).

**Table 3:** Sociodemographic and clinical characteristics of patients hospitalized who underwent a bioprosthetic surgical aortic valve replacement (SAVR) in Spain from 2001 to 2015.

	2001-05	2006-10	2011-15	Total	Trend
Number of SAVRs	7384	13848	19649	40881	
Age. mean (SD)	73.2 (7.76)	74.09 (7.99)	75.08 (7.59)	74.41 (7.79)	<0.001
18-59 age group, n (%)	322 (4.36)	588 (4.25)	655 (3.33)	1565 (3.83)	<0.001
60-69 age group, n (%)	1139 (15.43)	1956 (14.12)	2754 (14.02)	5849 (14.31)	<0.001
70-79 age group, n (%)	4871 (65.97)	8428 (60.86)	10698 (54.45)	23997 (58.7)	<0.001
≥80 age group, n (%)	1052 (14.25)	2876 (20.77)	5542 (28.2)	9470 (23.16)	<0.001
Female sex, n (%)	3249 (44)	6112 (44.14)	8503 (43.27)	17864 (43.7)	0.016
CCI index. mean (SD)	0.85 (0.92)	0.97 (0.96)	1.04 (0.99)	0.98 (0.97)	<0.001
T2DM, n (%)	1443 (19.54)	3418 (24.68)	5437 (27.67)	10298 (25.19)	<0.001
COPD, n (%)	743 (10.06)	1364 (9.85)	1820 (9.26)	3927 (9.61)	0.020
Peripheral vascular disease, n (%)	786 (10.64)	1669 (12.05)	2358 (12)	4813 (11.77)	<0.001
Chronic kidney disease, n (%)	421 (5.7)	1132 (8.17)	2234 (11.37)	3787 (9.26)	<0.001
Cerebrovascular disease, n (%)	292 (3.95)	618 (4.46)	1151 (5.86)	2061 (5.04)	<0.001
Congestive heart failure, n (%)	1090 (14.76)	2125 (15.35)	3244 (16.51)	6459 (15.8)	<0.001
Atrial fibrillation, n (%)	2384 (32.29)	4878 (35.23)	7327 (37.29)	14589 (35.69)	<0.001
Coronary artery disease, n (%)	2553 (34.57)	5170 (37.33)	7175 (36.52)	14898 (36.44)	<0.001
Pulmonary hypertension, n (%)	399 (5.4)	876 (6.33)	1165 (5.93)	2440 (5.97)	0.031
CABG, n (%)	1798 (24.35)	3602 (26.01)	4774 (24.3)	10174 (24.89)	<0.001
Pacemaker implantation, n (%)	267 (3.62)	494 (3.57)	915 (4.66)	1676 (4.1)	<0.001
Emergency room, n (%)	1655 (22.41)	2927 (21.14)	4054 (20.63)	8636 (21.12)	<0.001
LOHS. mean (SD)	20.7 (17.33)	19.62 (17.58)	16.81 (15.4)	18.47 (16.6)	<0.001
IHM, n (%)	550 (7.45)	917 (6.62)	984 (5.01)	2451 (6)	<0.001

CABG=coronary artery bypass grafting; CCI=Charlson comorbidity index; COPD=chronic obstructive pulmonary disease; LOHS=length of hospital stay; IHM=in-hospital mortality; T2DM=type 2 diabetes mellitus

### Factors Associated with IHM (Table 4)

**Table 4:** Multivariable analysis of factors associated with in-hospital mortality among patients who underwent surgical aortic valve replacement (SAVR) according to the valve type.

		Both types	Mechanical	Bioprosthetic
		OR (95% CI)	OR (95% CI)	OR (95% CI)
Year	2001-03	1	1	1
	2004-06	0.97 (0.89-1.06)	0.93 (0.84-1.03)	1.06 (0.9-1.25)
	2007-09	0.89 (0.82-0.98)	0.89 (0.79-0.99)	0.92 (0.78-1.08)
	2010-12	0.71 (0.65-0.78)	0.74 (0.66-0.83)	0.7 (0.6-0.82)
	2013-15	0.58 (0.53-0.64)	0.58 (0.52-0.66)	0.59 (0.5-0.69)
Sex	Female	1.3 (1.23-1.38)	1.33 (1.23-1.43)	1.27 (1.16-1.38)
Age groups in years	18-59	1	1	1
	60-69	1.36 (1.22-1.51)	1.41 (1.26-1.59)	0.77 (0.59-1)
	70-79	2.35 (2.12-2.59)	2.68 (2.4-2.99)	1.07 (0.84-1.36)
	≥80	2.99 (2.67-3.36)	3.28 (2.84-3.79)	1.42 (1.11-1.82)
T2DM	Yes	0.75 (0.7-0.81)	0.77 (0.7-0.84)	0.73 (0.66-0.81)
Peripheral vascular disease	Yes	1.18 (1.09-1.28)	1.23 (1.12-1.36)	----
Chronic kidney disease	Yes	1.7 (1.56-1.85)	1.71 (1.51-1.92)	1.7 (1.5-1.91)
Cerebrovascular disease	Yes	1.77 (1.6-1.97)	2.02 (1.75-2.33)	1.53 (1.31-1.8)
Congestive heart failure	Yes	2.49 (2.34-2.65)	2.43 (2.24-2.64)	2.62 (2.39-2.88)
Atrial fibrillation	Yes	0.78 (0.73-0.82)	0.78 (0.72-0.84)	0.78 (0.72-0.86)
Coronary artery disease	Yes	1.33 (1.23-1.44)	1.3 (1.17-1.44)	1.38 (1.23-1.56)
Pulmonary hypertension	Yes	1.19 (1.07-1.32)	-	1.26 (1.08-1.47)
CABG	Yes	1.28 (1.17-1.39)	1.35 (1.21-1.51)	1.2 (1.06-1.36)
Pacemaker implantation	Yes	0.79 (0.68-0.92)	0.7 (0.56-0.86)	-
Emergency room	Yes	1.74 (1.64-1.84)	1.94 (1.8-2.1)	1.47 (1.34-1.61)
Mechanical valve type	Yes	1.67 (1.57-1.77)	NA	NA

CABG=coronary artery bypass grafting; T2DM=type 2 diabetes mellitus; OR=odds ratio obtained using logistic regression models; 95% CI=95% confidence intervals Only those variables that demonstrated a significant association are shown. NA=not applicable

The time trend analysis showed a significant decrease in IHM from 2007-09 to 2013-2015 in patients who underwent mechanical SAVR and from 2010-12 to the last period in patients who underwent bioprosthetic SAVR.

Regardless of the type of valve implanted, factors that increased IHM included advanced age, female sex, renal disease, cerebrovascular disease, congestive heart failure and coronary artery disease. Patients with peripheral vascular disease who underwent mechanical SAVR had 1.23 times the risk of dying compared to patients without this comorbidity (OR 1.23; 95% CI 1.12-1.36). The presence of pulmonary hypertension increased the risk of death in patients who underwent bioprosthetic SAVR (OR 1.26; 95% CI 1.08-1.47).

In both types of SAVR, concomitant CABG and emergency room admission were factors associated with a higher risk of death (Table 4).

Factors associated with a lower risk of death for both types of SAVR included type 2 diabetes mellitus (T2DM) and atrial fibrillation. Patients who underwent mechanical SAVR and received a pacemaker had lower risk of death (OR 0.7; 95% CI 0.56-0.86).

Finally, after adjusting for study variables, mechanical SAVR was associated with a significantly higher IHM (OR 1.67; 95% CI 1.57-1.77) than bioprosthetic SAVR in our study.

## DISCUSSION

In the past 15 years, there has been a substantial increase in the number of heart valve surgeries in Spain. This increase is in line with the trend observed in other European countries and in the United States^[3,6,10,11]^. A retrospective study found that the number of procedures increased from 47.5 to 88.9 per 100,000 Medicare beneficiaries between 2009 and 2015^[11]^. A large study based on national registry data in the Netherlands showed more than twice the number of SAVRs were reported in 1995 compared with 2010^[10]^. The authors concluded that this trend can be partly attributed to an increased prevalence of valvular heart disease and partly to an increased proportion of diseased patients that are diagnosed. Because the mean age of the patients has risen, both are likely to have played an important role^[3,10]^.

Previous studies have found that after the introduction of transcatheter aortic valve replacement (TAVR), a slight decline in the number of SAVRs was observed over time^[11-13]^. Silaschi et al.^[12]^ indicated that this may be explained by a substantial number of patients being eligible for both types of procedures and the consequence that this leads to a reduction in surgically treated patients. However, in a single high-volume Canadian center, the surgical volume of isolated SAVR increased since the introduction of TAVR^[14]^. In our study, we cannot analyze the effect of TAVR over the trends of SAVR because, although TAVR was introduced in Europe in 2008, adoption trends in Spain seemed to have a plateau between 2008 and 2012 in comparison with other European countries^[15]^.

As expected, a substantial reduction in the rate of mechanical valves implanted was seen, and we found an increase in the use of bioprosthetic valves. A study using NIS found an increase in bioprosthetic valve use from 37.7% in 1998-2001 to 63.6% in 2007-2011^[4]^. This trend has also been reported in other registries^[3,6,10,16]^ and suggested improved durability of biological prostheses, fewer neurological and functional complications and avoidance of permanent anticoagulation^[12,17]^. Lastly, technological advances such as the valve-in-valve transcatheter procedure provided new alternatives for reoperations in biological prostheses^[10]^.

As found in other studies, patients with coronary artery disease, atrial fibrillation and T2DM are more likely to receive bioprosthetic than mechanical valves^[4]^. Age plays a major role in bioprosthetic valve selection for patients with and without comorbidities, specifically in coronary artery disease patients requiring revascularization^[4]^. We observed that valve choice is influenced by age, with most patients aged 70 to 79 years receiving bioprosthetic valves, and patients with a lower mean age of 65.27 years receiving mechanical valves.

We found that IHM of all types of SAVR has significantly decreased over the last 15 years. Siregar et al.^[10]^ found that IHM for SAVR with or without CABG decreased significantly from 3.5% in 2007 to 2.4% in 2010. A similar trend was found for operative mortality in most other studies, which could reflect a combination of improved overall health care, healthier aging and gradual improvements in cardiac surgery over time^[3,16]^.

In our study, aortic valve replacement in patients with a bioprosthetic valve was associated with lower IHM than in patients with a mechanical valve, which is consistent with observational evidence. In the general population, bioprosthetic valves are associated with lower IHM compared with mechanical prostheses, which come at the cost of slightly higher rates of in-hospital complications^[4]^. Du et al.^[5]^ found that the risk of death at the time of surgery was 60% higher for mechanical-valve recipients compared with bioprosthetic valve recipients. Isaacs et al.^[4]^ found higher IHM among patients who received mechanical valves (5.2%), compared with bioprosthetic valves (4.4%).

As we expected, we found an increased risk of IHM with increasing age. Using the NIS database, Agarwall et al.^[18]^ reported that patients with advanced age and high surgical risk have increased IHM and the incidence of adverse neurologic events after SAVR.

Female sex is a factor associated with IHM in patients who underwent SAVR. In agreement with this finding, a study using the NIS data found that IHM was significantly higher in women than in men (5.6% vs. 4%)^[19]^. This could be related to the onset mechanism for cardiovascular disease, a delay in the presentation of valve problems and/or a later referral of women to cardiothoracic surgery^[20]^.

Chronic kidney disease, peripheral vascular disease, cerebrovascular disease and congestive heart failure are risk factors of IHM. However, the presence of T2DM and atrial fibrillation are associated with a lower risk of death for both types of SAVR. Halkos et al.^[21]^ found that diabetes was not associated with IHM (OR 0.86; 95% CI 0.49-1.50) after SAVR. The lower IHM in T2DM patients undergoing SAVR compared to nondiabetic patients might be multifactorial. Obesity is more prevalent in T2DM patients undergoing SAVR and this may have contributed to the decrease of IHM, as previously mentioned^[22]^.

We found a significantly higher IHM in patients who underwent bioprosthetic or mechanical SAVR performed with CABG than in those patients who did not undergo this procedure. Analysis of the German Aortic Valve Registry study showed that IHM was 1.7% for the SAVR group and 3.3% for the SAVR+CABG group, and this outcome measure remained constant between 2011 and 2015 in both groups^[6]^.

Several previous studies have shown increased preoperative risk/comorbidities and rate of postoperative complications in patients requiring permanent pacemaker after SAVR^[23,24]^. We found that pacemaker implantation reduces the risk of IHM after mechanical SAVR. In agreement with this finding, a study using NIS data found that permanent pacemaker implantation following SAVR was associated with lower IHM^[25]^. However, Greason et al.^[24]^ found a significant association of early pacemaker implantation with death [HR 1.49 (95% CI 1.20-1.84)].

We have found longer LOHS in our country compared to previous studies^[6,16,19]^. We believe that differences in patient baseline characteristics (comorbidities and age) and in the use of concomitant procedures, such as CABG, could partly explain this result. It is also remarkable that SNHDD only provides the date of admission and the date of discharge, so the LOHS in our investigation includes pre and postoperative time. This fact may contribute to our longer LOHS, when compared to other investigations, which only analyzed postoperative time^[16]^.

There are some points that should be taken into consideration when interpreting the results of the present study. Our data source was SNHDD, an administrative database that contains discharge data for hospitalizations in Spain and uses the information the physician has included in the discharge report^[7]^. Coding practices, as well as coding errors, may differ between individual physicians and institutions. Thus, our results are subject to several potential biases, including differences in the capture of adverse outcomes across hospitals along the study period.

The SNHDD only provides the patient’s vital status at discharge, but not the cause of death, so this relevant information is not included in our study. Furthermore, for confidentiality reasons, hospital names and characteristics are not included in the SNHDD, so this information could not be analyzed. Not all hospitals may have the same expected result, and the more specialized centers may have better outcomes, as described earlier^[26]^. However, we think this would not affect our main results because we included all Spanish hospitals, beside their characteristics, over the 15-year period.

Finally, there is a lack of information on previous cardiac surgery (PCS) that may be a risk factor for operative mortality in patients undergoing aortic valve replacement. However, previous studies have found that, after propensity matching of PCS patients with first-time surgery, previous surgery was not a predictor of operative mortality nor long-term survival in patients undergoing isolated aortic valve replacement^[27]^.

## CONCLUSION

The results of this study provide a comprehensive overview of valve surgery trends and outcomes in Spain. The number of SAVRs performed in Spain has increased since 2001. The use of mechanical SAVR has decreased and the use of bioprosthetic valves has increased over time. IHM has decreased over time, despite a concomitant increase in age and comorbidity of patients over the same period. Higher IHM rates in patients were associated with increased age, female sex, presence of comorbidities, and concomitant CABG. Remarkably, patients who underwent mechanical SAVR had higher IHM than those who underwent bioprosthetic SAVR.

**Table:** 

Authors' roles & responsibilities	
RJG	Substantial contributions to the conception or design of the work; or the acquisition, analysis, or interpretation of data for the work; drafting the work or revising it critically for important intellectual content; agreement to be accountable for all aspects of the work in ensuring that questions related to the accuracy or integrity of any part of the work are appropriately investigated and resolved; final approval of the version to be published
NPF	Substantial contributions to the conception or design of the work; or the acquisition, analysis, or interpretation of data for the work; drafting the work or revising it critically for important intellectual content; agreement to be accountable for all aspects of the work in ensuring that questions related to the accuracy or integrity of any part of the work are appropriately investigated and resolved; final approval of the version to be published
JMD	Substantial contributions to the conception or design of the work; or the acquisition, analysis, or interpretation of data for the work; drafting the work or revising it critically for important intellectual content; agreement to be accountable for all aspects of the work in ensuring that questions related to the accuracy or integrity of any part of the work are appropriately investigated and resolved; final approval of the version to be published
VHB	Substantial contributions to the conception or design of the work; or the acquisition, analysis, or interpretation of data for the work; drafting the work or revising it critically for important intellectual content; agreement to be accountable for all aspects of the work in ensuring that questions related to the accuracy or integrity of any part of the work are appropriately investigated and resolved; final approval of the version to be published
MMB	Substantial contributions to the conception or design of the work; or the acquisition, analysis, or interpretation of data for the work; drafting the work or revising it critically for important intellectual content; agreement to be accountable for all aspects of the work in ensuring that questions related to the accuracy or integrity of any part of the work are appropriately investigated and resolved; final approval of the version to be published
IJT	Substantial contributions to the conception or design of the work; or the acquisition, analysis, or interpretation of data for the work; drafting the work or revising it critically for important intellectual content; agreement to be accountable for all aspects of the work in ensuring that questions related to the accuracy or integrity of any part of the work are appropriately investigated and resolved; final approval of the version to be published
JMMY	Substantial contributions to the conception or design of the work; or the acquisition, analysis, or interpretation of data for the work; drafting the work or revising it critically for important intellectual content; agreement to be accountable for all aspects of the work in ensuring that questions related to the accuracy or integrity of any part of the work are appropriately investigated and resolved; final approval of the version to be published
ALA	Substantial contributions to the conception or design of the work; or the acquisition, analysis, or interpretation of data for the work; drafting the work or revising it critically for important intellectual content; agreement to be accountable for all aspects of the work in ensuring that questions related to the accuracy or integrity of any part of the work are appropriately investigated and resolved; final approval of the version to be published
